# Un syndrome d’insuffisance hépatocellulaire révélant une leucémie à grands lymphocytes granuleux type T: à propos d’un cas marocain et revue de la littérature

**DOI:** 10.11604/pamj.2019.34.119.19715

**Published:** 2019-10-29

**Authors:** Amine Benmoussa, Leila Oussaih, Illias Tazi

**Affiliations:** 1 Service d'Hématologie CHU Mohammed VI, Marrakech, Maroc

**Keywords:** La leucémie à grands lymphocytes granuleux T, un syndrome d’insuffisance hépatocellulaire, neutropénie, pronostic, traitement, T-cell large granular lymphocyte (LGL) leukemia, hepatocellular failure, neutropenia, prognosis, treatment

## Abstract

La leucémie à grands lymphocytes granuleux type T (T-LGL) fait partie des hémopathies lymphoïdes à cellules T/NK (natural killer) matures dans la dernière classification de l'organisation mondiale de santé (OMS) 2016. La T-LGL est caractérisée par un phénotype T CD3+ et un réarrangement du gène TCR (récepteur de lymphocyte T). C'est une entité rare qui s'observe essentiellement en occident. Nous rapportons le cas d'un patient de 60 ans, d'origine marocaine, qui présente depuis 1 mois un ictère généralisé avec des pétéchies diffuses et altération de l'état général, l'examen clinique initial objective une ascite de moyenne abondance et hépatosplénomégalie, l'examen paraclinique montre des signes biologiques d'insuffisance hépatocellulaire avec une neutropénie et thrombopénie. Le diagnostic de la T-LGL est retenu par l'étude cytologique et immunophénotyique du sang médullaire ainsi que la détection de réarrangement de TCR à la biologie moléculaire. Le patient est mis sous cyclophosphamide avec traitement symptomatique. L'évolution est fatale avec décès du patient 3 mois après le diagnostic par un choc septique sur un état de cachexie. La T-LGL est d'évolution très souvent indolente et son tableau clinique initial au diagnostic est généralement modéré et d'installation progressive et ce n'est pas le cas chez notre patient. Un syndrome d'insuffisance hépatocellulaire est exceptionnellement inaugural d'une leucémie à grands lymphocytes granuleux type T.

## Abstract (en)

In the last classification of the World Health Organization (WHO) 2016, T-cell large granular lymphocyte (LGL) leukemia is a type of mature natural killer T (NKT) cell lymphatic hematologic disease. T-LGL leukemia is characterized by CD3+ T-cell phenotype and TCR gene rearrangement (T lymphocyte receptor). It is a rare disease, mainly affecting people living in the Western world. We report the case of a 60-year old Moroccan patient presenting with 1-month history of generalized jaundice with diffuse petechiae and impaired general condition. Initial clinical examination showed ascites of average abundance and hepatosplenomegaly; paraclinical examination objectified hepatocellular failure with neutropenia and thrombocytopenia. The diagnosis of T-LGL was retained based on cytological and immunophenotypic study of bone marrow blood as well as on the detection of TCR rearrangement using molecular test. The patient received cyclophosphamide associated with symptomatic treatment. The patient died within three months of the diagnosis due to septic shock with cachexia. T-LGL often has an indolent behavior and initial clinical status at diagnosis is generally moderate, with progressive onset but that was not so for our patient. Hepatocellular failure exceptionally results in T-cell large granular lymphocyte (LGL) leukemia.

## Introduction

La leucémie à grands lymphocytes granuleux T (T-LGL) est une pathologie rare secondaire à une lymphoprolifération clonale de cellules T de profil CD3+. Il s'agit d'une pathologie au spectre hétérogène incluant des formes indolentes ou paucisymptomatiques d'évolution chronique et des formes agressives. Les leucémies à LGL CD3+ se caractérisent par une neutropénie et/ou des manifestations auto-immunes (essentiellement la polyarthrite rhumatoïde). La physiopathologie de ces lymphoproliférations LGL repose sur un facteur déclenchant viral (EBV: Epstein-Barr virus ou HTLV: Human T-cell Leukemia Virus) ou auto-immun, une dérégulation de l'apoptose et une activation cytokinique. Le diagnostic repose sur la mise en évidence du caractère clonal de la prolifération Lymphocytaire. Le traitement des leucémies à LGL CD3+ n'est indiqué que dans les formes symptomatiques (complications infectieuses et/ou auto-immunes) [1, 2].

## Patient et observation

Il s'agit d'un patient âgé de 60 ans, sans antécédents pathologiques particuliers, qui présente depuis un mois, un ictère généralisé avec des pétéchies disséminées dans le corps entier, le tout évolue dans un contexte d'altération de l’état général (amaigrissement chiffré à 6kg avec asthénie et anorexie). L'examen clinique initial trouve un patient conscient avec un score de Glasgow à 15/15^ème^, apyrétique T: 37°C, une tension artérielle à 12/7cmHg, une saturation en Oxygène à 100%, une fréquence cardiaque à 65 bat/min, des conjonctives décolorées, des pétéchies diffuses, hépatomégalie avec une flèche hépatique à 16cm, une splénomégalie à 3cm de débord costal, pas d'adénopathies périphériques, une ascite de moyenne abondance, le reste de l'examen somatique est normal. Le bilan biologique montre à l'hémogramme une anémie normocytaire normochrome arégénérative avec Hb (hémoglobine) à 6.5g/dl, volume globulaire moyen (VGM) à 89 femtolitre, teneur corpusculaire en hémoglobine (TCMH) à 29 et réticulocytes à 95 10^9^/L, une thrombopénie avec un nombre de plaquettes à 30 10^9^/L, avec des globules blancs à 8 10^9^/L, des polynucléaires neutrophiles à 0,4 10^9^/L, et des lymphocytes à 5 10^9^/L. Le frottis sanguin montre la présence des grands lymphocytes granuleux. Le myélogramme est riche avec la présence de quelques mégacaryocytes (R+++ M++) et hyperplasie lymphoïde à 40% avec des grands lymphocytes granuleux. L'immunophénotypage du sang périphérique montre des cellules lymphoïdes granuleux exprimant le CD7+, CD8+, CD3+, CD45+, et n'exprimant pas le CD4-, CD10-, CD19-, CD20-, CD56-, cet aspect est en faveur de leucémie à LGL. Le réarrangement TCR est positif à la biologie moléculaire.

Le reste de bilan objective des signes d'insuffisance hépatique avec une cytolyse hépatique, une alanine amino-transférase (ALAT) à 280 UI/L et une aspartateamino-transférase (ASAT) à 255 UI/L, lacticodéshydrogénase (LDH) est élevée à 1200 UI/L, albumine: 19g/l, bilirubine conjuguée augmentée à 60mg/l, la bilirubine non conjuguée est normale à 1mg/l, phosphatase alcaline augmentée: 500 U/L, urée à 0,25g/l, créatinine à 10mg/l, fibrinogène: 1,1g/l, taux de prothrombine: 60 pour cent; le temps de céphaline activé est à 40 sec, le test de coombs direct est négatif, les sérologies VIH, VHC, EBV, VHB sont négatives, le bilan immunologique est négatif (Ac AntiDNA, Ac Antimuscle lisse, Ac Antinucléaires, Ac Anti nucléocytoplasmique, facteur rhumatoïde). Le bilan radiologique montre une hépatomégalie à 16cm avec splénomégalie à 19cm et ascite de moyenne abondance au scanner thoraco-abdomino-pelvien. La fibroscopie œsogastroduodénale montre un aspect de gastrite chronique. La ponction-biopsie hépatique objective des lesions hépatocytaires nécrotico inflammatoires. Le patient est mis sous traitement symptomatique à base des transfusions en culots plaquettaires et globulaires à répétition en fonction des chiffres de l'hémoglobine et des plaquettes dans les hémogrammes de contrôle avec des perfusions de l'albumine et de plasma frais congelé en fonction des chiffres de l'albumine et de bilan d'hémostase. Le traitement spécifique à base de cyclophosphamide à la dose de 100mg/j est démarré dès l'établissement de diagnostic. L'évolution est marquée par l'amélioration de cytopénie et la diminution des besoins transfusionnels après 1 mois de traitement par cyclophophamide mais le malade garde toujours une anorexie avec une légère prise de poids, le tableau clinique se complique 3 mois plus tard par un état de choc septique, le patient est décédé au Service de Réanimation.

## Discussion

La leucémie à grands lymphocytes granuleux (LGL) à cellules T est une prolifération clonale de cellules T cytotoxiques (CD8+), se caractérise cliniquement par une neutropénie, une anémie et/ou une thrombopénie et une légère lymphocytose, elle peut s'associer à plusieurs désordres auto-immuns [1]. Comme le cas présent, notre patient présente une hépatite auto-immune avec syndrome d'insuffisance hépatocellulaire. Les leucémies LGL T et NK (natural killer) représentent seulement 2 à 5% des syndromes lymphoprolifératifs en Occident. Cette prévalence augmente jusqu'à 9% en Asie. Les leucémies LGL T CD3+ représentent 85% des leucémies LGL (15% des LGL sont des leucémies à cellules NK). Elles affectent essentiellement les adultes de 55 à 60 ans, l'âge médian au diagnostic est de 59 ans, elles touchent les 2 sexes de façon égale avec sex-ratio à 1 [2]. Elles sont classiquement associées à une maladie auto-immune surtout polyarthrite rhumatoïde et rarement une hépatite auto-immune, comme le cas de notre patient. Les signes cliniques sont liés aux cytopénies et aux maladies auto-immunes associées. Ils sont peu fréquents au diagnostic (un tiers des patients asymptomatique). L'altération de l'état général (fièvre, amaigrissement, sueurs profuses) est retrouvée dans 20 à 30% des cas. L'examen clinique est souvent peu contributif. La splénomégalie est observée dans moins de 50% des cas, l'hépatomégalie dans un quart des cas. Les adénopathies sont rarement présentes. Notre patient présente une altération de l'état général avec hépatosplénomégalie sans adénopathies. La neutropénie chronique, présente dans plus de 80% des cas, pouvant être responsable d'un tableau sévère lors d'agranulocytose fébrile (< 0,5g/l), avec risque d'infections récidivantes mettant en jeu le pronostic vital. Notre patient présente une agranulocytose au diagnostic [3]. Les maladies auto-immunes sont associées à la leucémie à LGL T dans moins de 50% des cas. Il s'agit majoritairement de la polyarthrite rhumatoïde (25 à 30% des cas) qui peut la précéder ou la révéler. D'autres maladies auto-immunes ont été rapportées (Sd de Goujerot-Sjögrenprimaire, lupus érythémateux disséminé, maladie cœliaque, sclérodermie, thyroïdites) [4, 5]. La Leucémie T LGL est rarement révélée ou compliquée par une hépatite auto-immune réalisant un tableau d'insuffisance hépatocellulaire. Dans le cas présent, l'hépatite auto-immune compliquée de syndrome d'insuffisance hépatocellulaire a révélé la leucémie T LGL.

Le diagnostic repose sur l'étude cytologique, immunophénotypique et moléculaire du sang périphérique ou médullaire. L'étude cytologique montre de grandes cellules (15 à 20mm de diamètre) avec un cytoplasme abondant contenant un noyau à large nucléole et des granulations azurophiles facilement visualisées par la coloration au May Grunwald Giemsa. Cet aspect n'est pas toujours typique et les granules cytoplasmiques peuvent être absents [6]. L'étude immunophénotypique montre des cellules lymphoïdes granuleuses avec un phénotype T de cellules postthymiques matures: CD3+/TCRab+/CD4-/CD8+/CD45RO-. Elles se caractérisent par le cluster CD57+ spécifique des cellules effectrices issues d'une population de cellules mémoires CD57-. Quelques formes de phénotypes inhabituels sont décrites: CD3>+/CD4+/CD8- ou CD<3+/CD4+/CD8+. L'étude moléculaire confirme le caractère monoclonal qui permet de faire la distinction avec les expansions LGL T réactionnelles. Elle est réalisée à partir de l'étude du réarrangement du gène codant pour le TCR (T cell receptor) par “polymerase chain reaction (PCR)” sur acide désoxyribonucléique (ADN) génomique [3]. Le diagnostic chez notre patient est confirmé par l'étude cytologique et immunophénotypique du sang médullaire. Le pronostic de cette pathologie dépend des complications infectieuses et immunologiques. La médiane de survie est supérieure à dix ans [7]. Dans le cas présent l'évolution est rapidement défavorable, avec le décès du patient 3 mois après le diagnostic. Le traitement de la leucémie T LGL n'est indiqué que lorsqu'il existe une neutropénie inférieure à 0.5g/l, ou supérieure à 0.5g/l compliquée d'infections récurrentes ou anémie symptomatique ou la présence d'une maladie auto-immune associée [8]. Le traitement spécifique se base essentiellement sur leméthotrexate, la ciclosporine, et la cyclophosphamide. Le méthotrexate est la molécule la plus utilisée en cas de neutropénie a la posologie hebdomadaire de 10mg/m^2^, le taux de réponse est d'environ 55% [9]. La ciclosporine est utilisée surtout en cas d'érythroblastopénie ou en deuxième ligne après le méthotrexate avec des taux de réponse de l’ordre de 50% [10]. Le cyclophosphamide per os à la posologie de 50 à 100mg, peut être utilisée en première ligne ou en cas d'échec des autres thérapeutiques avec un taux de réponse globale de 66%. La durée de ce traitement est de préférence limitée à 12 mois du fait des effets secondaires [11]. En cas d'absence de réponse à ces trois immunosuppresseurs de référence (méthotrexate, cyclophosphamide et ciclosporine), les alternatives reposent sur les analogues puriques (pentostatine, fludarabine et cladribine) [12].

## Conclusion

La leucémie T à LGL est une pathologie rare, d'évolution souvent indolente, les formes symptomatiques doivent faire initier un traitement adapté, du fait des complications lourdes qu'elles peuvent causer. La prise en charge n'est pas codifiée d'où l'intérêt d'autres études afin de standardiser le traitement et améliorer le pronostic en cas d'atteinte sévère.

## Conflits d’intérêts

Les auteurs ne déclarent aucun conflit d'intérêts.
